# Effects of anthracycline, cyclophosphamide and taxane chemotherapy on QTc measurements in patients with breast cancer

**DOI:** 10.1371/journal.pone.0196763

**Published:** 2018-05-03

**Authors:** Pedro Veronese, Denise Tessariol Hachul, Mauricio Ibrahim Scanavacca, Ludhmila Abrahão Hajjar, Tan Chen Wu, Luciana Sacilotto, Carolina Veronese, Francisco Carlos da Costa Darrieux

**Affiliations:** 1 Clinical Unit of Arrhythmia and Pacing, Instituto do Coracao (InCor), Hospital das Clinicas HCFMUSP, Faculdade de Medicina, Universidade de Sao Paulo - São Paulo, São Paulo, Brazil; 2 Oncology Department, Cancer Institute (ICESP), Hospital das Clinicas HCFMUSP, Faculdade de Medicina, Universidade de Sao Paulo –São Paulo, São Paulo, Brazil; 3 Clinical Department, The University of North Carolina at Chapel Hill – Chapel Hill, North Carolina, United States of America; University of São Paulo, BRAZIL

## Abstract

**Aim:**

Acute and subacute cardiotoxicity are characterized by prolongation of the corrected QT interval (QTc) and other measures derived from the QTc interval, such as QTc dispersion (QTdc) and transmural dispersion of repolarization (DTpTe). Although anthracyclines prolong the QTc interval, it is unclear whether breast cancer patients who undergo the ACT chemotherapy regimen of anthracycline (doxorubicin: A), cyclophosphamide (C) and taxane (T) may present with QTc, QTdc and DTpTe prolongation.

**Methods:**

Twenty-three consecutive patients with breast cancer were followed prospectively during ACT chemotherapy and were analyzed according to their QT measurements. QTc, QTdc and DTpTe measurements were determined by a 12-lead electrocardiogram (EKG) prior to chemotherapy (baseline), immediately after the first phase of anthracycline and cyclophosphamide (AC) treatment, and immediately after T treatment. Serum troponin and B-type natriuretic peptide (BNP) levels were also measured.

**Results:**

Compared to baseline values, the QTc interval was significantly prolonged after the AC phase (439.7 ± 33.2 ms vs. 472.5 ± 36.3 ms, p = 0.001) and after T treatment (439.7 ± 33.2 ms vs. 467.9 ± 42.6 ms, p < 0.001). Troponin levels were elevated after the AC phase (23.0 pg/mL [min-max: 6.0–85.0] vs. 6.0 pg/mL [min-max: 6.0–22.0], p < 0.001) and after T treatment (25.0 pg/mL [min-max: 6.0–80.0] vs. 6.0 pg/mL [min-max: 6.0–22.0], p < 0.001) compared to baseline values.

**Conclusion:**

In this prospective study of patients with non-metastatic breast cancer who underwent ACT chemotherapy, significant QTc prolongation and an elevation in serum troponin levels were observed.

## Introduction

In addition to skin cancer, breast cancer is the most common type of cancer in women. Advancements in chemotherapy strategies for breast cancer patients have contributed to high remission rates. However, chemotherapy-related cardiotoxicity has been a subject of intensive research all over the world. Among other findings, the research characterized acute and subacute cardiotoxicity by acute coronary artery syndrome, pericarditis, myocarditis, cardiac arrhythmias, or QT interval prolongation [1, 2]. Such cardiac changes may occur during chemotherapy treatment or even up to two weeks after the conclusion of chemotherapy [3]. Chronic cardiotoxicity may result in a reduced left ventricle ejection fraction (LVEF), heart failure, or death [4].

Anthracyclines, which are routinely used to treat breast cancer, may induce acute, subacute, and chronic cardiotoxicity, and the latter is usually caused by cumulative doses of 400 mg/m^2^ or greater [5,6].

Corrected QT interval (QTc) prolongation and other measures derived from the QTc interval, such as the dispersion of the QTc (QTdc) and the transmural dispersion of repolarization (DTpTe), are markers of potentially lethal arrhythmias, e.g., *torsades de pointes* [7,8].

Previous studies in patients with non-Hodgkin lymphoma who received anthracyclines have demonstrated QTc interval prolongation and increased QTdc [9,10]. However, the chemotherapeutic doses used in this population were much higher than those used to treat breast cancer patients. Similar findings were observed in breast cancer patients with HER2 protein expression who were consequently exposed to both anthracycline and trastuzumab [11].

Although anthracyclines prolong the QTc interval, [2] it remains unclear whether breast cancer patients who were exposed specifically to the chemotherapy regimen with anthracycline (A; doxorubicin), cyclophosphamide (C) and taxane (T; paclitaxel), known as the ACT regimen, may present with QTc, QTdc and DTpTe prolongation.

The primary objective of this study was to evaluate the effect of the ACT chemotherapy regimen on the QTc interval in the early phase of treatment. The secondary objectives were 1) to evaluate the effect of ACT chemotherapy on the QTdc and DTpTe values; 2) to assess any changes in cardio-specific biomarkers, such as troponin and B-type natriuretic peptide (BNP); and 3) to assess the clinical manifestations of cardiotoxicity, including the presence of cardiac arrhythmias, congestive heart failure (CHF), angina and cardiovascular death in patients with breast neoplasms who were undergoing ACT chemotherapy.

## Methods

### Study patients

Twenty-seven consecutive patients diagnosed with non-metastatic breast cancer were selected from August 2015 to February 2017 and were all under treatment at the Cancer Institute of the State of São Paulo (ICESP). The patients were undergoing adjuvant or neoadjuvant chemotherapy based on the Institute’s standardized protocol for ACT chemotherapy, including anthracycline (doxorubicin), cyclophosphamide and taxane (paclitaxel) infusion. None of the patients had any known structural heart diseases or previous cardiovascular events. None of the patients had received previous chemotherapy or radiotherapy. All the patients were prospectively followed up and had a 12-lead electrocardiogram (EKG) recorded prior to chemotherapy initiation (baseline), immediately after the end of the first phase of chemotherapy (anthracycline and cyclophosphamide [AC] phase), and immediately after the end of the second phase (final) with paclitaxel (T). Serum troponin, BNP, and electrolytes (potassium, magnesium, and calcium) were measured at baseline, immediately after the AC phase of chemotherapy, and immediately after the T phase of chemotherapy.

The Scientific Committee of Instituto do Coração (InCor-HCFMUSP) and the Institutional Review Board (CAPPesq) of the *Hospital das Clínicas da Faculdade de Medicina da Universidade de São Paulo* (HCFMUSP) approved the study protocol. Written informed consent was obtained from each patient, and the study was conducted in accordance with the Declaration of Helsinki.

### Electrocardiography and toxicity assessments

All patients underwent an EKG soon after treatment. EKGs were recorded with a portable digital electrocardiograph (TEB C 30^+^, TEB Ltda, São Paulo, Brazil) with 10-sec recordings at a 25 mm/sec speed and a standard voltage of 1.0 mV (10 mm). The QT interval was manually measured using the tangent method from the beginning of the QRS complex to the end of the T wave from all 12 leads [11,12]. Whenever the end of the T wave could not be determined in any given lead, this lead was excluded from the analysis. The QTc interval was calculated in lead V5 using Bazett’s formula (QTc = QT interval / √RR interval). QTc values greater than 460 ms for women were considered abnormal [13,14]. QTdc was manually calculated by the difference between the longest and the shortest QTc intervals measured across the 12 leads. Whenever the end of the T wave could not be determined in a lead, this lead was excluded from the analysis. QTdc values greater than or equal to 50 ms were considered abnormal. DTpTe was manually calculated by the difference between the greatest and the smallest Tpeak to Tend (TpTe) interval measured across the 12 leads [13,14]. All measurements were made by the same cardiologist who was blinded to the patients’ data, and the measurements were later confirmed by a second cardiologist. Occasional disagreements were resolved by consensus.

Clinical evaluations of the patients were performed during clinical visits; determination of cardiac arrhythmias relied on symptoms and the data from the resting EKGs.

Troponin I levels were determined by a sandwich immunoassay in three stages using direct chemiluminescence technology and consistent quantities of two monoclonal antibodies. An adjuvant reactant was included to reduce nonspecific binding. For this purpose, the ADVIA Centaur^®^ Tnl-Ultra^®^ commercial kit (Siemens Healthcare Diagnostics, Tarrytown, NY, USA) was used with an automated detection system from the same manufacturer. The results are presented in pg/mL with a 99% detection threshold of 6 pg/mL.

Serum concentrations of BNP were obtained using a sandwich immunoassay in two stages using direct chemiluminescence technology and consistent quantities of two monoclonal antibodies. For this purpose, the ADVIA Centaur^®^ Tnl-Ultra^®^ commercial kit (Siemens Healthcare Diagnostics, Tarrytown, NY, USA) was used with an automated detection system from the same manufacturer. The results are presented in pg/mL, and the reference value for normal BNP serum concentration is below 100 pg/mL.

The ACT chemotherapy regimen consisted of doxorubicin, cyclophosphamide, and paclitaxel. This regimen was divided in two phases. The first phase comprised four cycles of AC (doxorubicin + cyclophosphamide) infused at intervals of 21 days. The total duration of the first (AC) phase was 63 days as follows: every 21 days, patients received an IV bolus infusion of 60 mg/m^2^ doxorubicin, 600 mg/m^2^ cyclophosphamide, and 20 mg dexamethasone. The second (T) phase lasted 8 weeks with weekly IV infusions of 100 mg/m^2^ paclitaxel and 8 mg dexamethasone. All patients received ondansetron before infusion of chemotherapeutic agents.

### Statistical analysis

Qualitative characteristics of the women undergoing chemotherapy are described using absolute and relative frequencies, and quantitative characteristics are described using summarized measures (mean and standard deviation are used for parametric variables; median, minimum, and maximum values are used for non-parametric variables).

The values of the EKG parameters are reported using summarized measures at each evaluation timepoint. The values at different timepoints were compared using ANOVAs with repeat measures followed by multiple Bonferroni comparisons, except for the comparisons of troponin and BNP levels where the Friedman test was used followed by non-parametric multiple comparisons for longitudinal data, when necessary.

The significance level applied for all statistical tests was 5%.

## Results

Twenty-seven consecutive female patients with breast neoplasms were evaluated. Two patients discontinued the research protocol, and another two were excluded from the study due to metastatic disease progression during the follow-up period. Thus, 23 patients completed the study. The mean follow-up duration was 8 months.

Demographics, clinical characteristics, and laboratory results of the 23 patients are shown in Table 1. None of the patients showed any evidence of structural heart disease at baseline, and nine patients exhibited stage I hypertension. All electrolyte levels were normal before and after the treatment.

**Table 1:** The demographic, clinical, and laboratory characteristics of all female study patients.

Variables	
Patients—n	23
Female—n (%)	23 (100)
Age—years (SD)	50 (9)
High blood pressure—n (%)	9 (39)
Diabetes mellitus—n (%)	2 (8)
Dyslipidemia—n (%)	2 (8)
Beta blocker—n (%)	3 (13)
SSRI—n (%)	3 (13)
ACE inhibitor—n (%)	4 (17)
ARB—n (%)	5 (21)
Calcium channel blocker—n (%)	2 (8)
Diuretic—n (%)	3 (13)
Metformin—n (%)	2 (8)
LVEF—% mean (SD)	65 (2.9)

SSRI: selective serotonin reuptake inhibitor, ACE: angiotensin-converting enzyme, ARB: angiotensin receptor blocker, LVEF: left ventricular ejection fraction, and SD: standard deviation.

The three patients who were previously on beta blockers (BBs) were maintained on the same dose of medication throughout the EKG measurements. The same occurred with three patients who were on selective serotonin reuptake inhibitors (SSRIs).

QTc interval measurements were significantly increased after the first (AC) phase of chemotherapy compared to the baseline values (472.5 ± 36.3 ms vs. 439.7 ± 33.2 ms; p = 0.001). There was also a significant increase in the QTc interval after the final (T) phase compared to baseline (467.9 ± 42.6 ms vs. 439.7 ± 33.2 ms; p < 0.001), as shown in Table 2. In our series, nine patients (39, 14%) presented with a QTc interval > 500 ms, but none of them suffered life-threatening arrhythmias (See S1 Table).

**Table 2:** Comparison of the QTc measurements.

QTc (ms)	Mean	SD	p-value
Initial values	439.7	33.2	
After AC	472.5	36.3	0.001*
After T	467.9	42.6	< 0.001*

ANOVA with repeated measurements followed by Bonferroni multiple comparisons.

*A two-tailed p-value of < 0.05 was considered statistically significant compared with initial values of QTc.

AC: anthracycline (doxorubicin) and cyclophosphamide, T: taxane (paclitaxel), SD: standard deviation, and QTc: corrected QT interval.

The QTdc was not significantly different (p = 0.20) compared to baseline neither after the AC phase (46.8 ± 28.0 ms vs. 57.0 ± 19.3 ms) nor at the end of chemotherapy (46.8 ± 28.0 ms vs. 54.0 ± 16.8 ms). The DTpTe was also not significantly different (p = 0.89) compared to baseline neither after the AC phase (33.5 ± 15.0 ms vs. 32.6 ± 12.1 ms) nor at the end of chemotherapy (33.5 ± 15.0 ms vs. 34.4 ± 10.4 ms). The results are shown in Table 3.

**Table 3:** Comparison of the QTdc and DTpTe measurements.

Variables	Mean	SD	p-value
**QTdc (ms)**			0.20
Initial values	46.8	28.0	
After AC	57.0	19.3	
After T	54.0	16.8	
**DTpTe (ms)**			0.89
Initial values	33.5	15.0	
After AC	32.6	12.1	
After T	34.4	10.4	

ANOVA with repeated measurements followed by Bonferroni multiple comparisons.

AC: anthracycline (doxorubicin) and cyclophosphamide, T: taxane (paclitaxel), QTdc: QTc dispersion, DTpTe: transmural dispersion of repolarization, and SD: standard deviation.

Serum BNP levels were not significantly different (p = 0.43) compared to baseline neither after the AC phase (13.0 pg/mL [min-max: 3.0–81.0] vs. 12.0 pg/mL [min-max: 5.0–103.0]) nor at the end of chemotherapy (13.0 pg/mL [min-max: 3.0–81.0] vs. 12.0 pg/mL [min-max: 2.0–45.0]).

Regarding troponin, there was a statistically significant increase in the serum values compared to the baseline values both after the AC phase (6.0 pg/mL [min-max: 6.0–22.0] vs. 23.0 pg/mL [min-max: 6.0–85.0], p < 0.001) and at the end of chemotherapy (6.0 pg/mL [min-max: 6.0–22] vs. 25.0 pg/mL [min-max: 6.0–80.0], p < 0.001), as shown in Table 4.

**Table 4:** Comparison of the troponin and BNP measurements.

Variables	Median	Min-max	p-value
**Troponin (pg/mL)**			
Initial values	6.0	06.0–22.0	
After AC	23.0	06.0–85.0	< 0.001*
After T	25.0	06.0–80.0	< 0.001*
**BNP (pg/mL)**			0.43
Initial values	13.0	03.0–81.0	
After AC	12.0	05.0–103.0	
After T	12.0	02.0–45.0	

Friedman test followed by multiple non-parametric comparisons for longitudinal data.

*A two-tailed p-value of < 0.05 was considered statistically significant compared with initial values.

AC: anthracycline (doxorubicin) and cyclophosphamide, T: taxane (paclitaxel), BNP: B-type natriuretic peptide, and min-max: minimum to maximum.

During the clinical follow-up period, no cases of death, angina, CHF, or cardiac arrhythmia were detected. The main symptoms observed were nausea, vomiting, weakness, and transient muscular pain associated with the chemotherapy.

## Discussion

The present prospective study contributes data on patients with breast neoplasms who underwent an ACT chemotherapy regimen (doxorubicin, cyclophosphamide, and paclitaxel), demonstrating prolongation of the QTc interval and an elevation in serum troponin levels among these patients.

Prolongation of the QT interval is one of the manifestations of acute and subacute chemotherapy-induced cardiotoxicity. Chemotherapy-induced cardiotoxicity, which can also manifest as the emergence of cardiac arrhythmias and alterations in ventricular repolarization, has not been fully elucidated. Such cardiac changes occur during chemotherapy treatment or even up to two weeks after the conclusion of chemotherapy [15]. Kitagawa et al. showed that patients with breast neoplasms who underwent a chemotherapy regimen with epirubicin, cyclophosphamide and 5-fluorouracil presented with QTc interval prolongation [16]. Tanriverdi et al. demonstrated that patients with breast neoplasms with HER2 expression who underwent a chemotherapy regimen with trastuzumab and anthracycline (epirubicin) also presented with QTc interval prolongation [11]. However, prolongation of the QTc interval in women with HER2-negative breast cancer undergoing the specific chemotherapy treatment regimen of doxorubicin, cyclophosphamide, and paclitaxel has never been studied until now.

Our study included only female patients with a mean age of 50 years old, where 13% used BBs and another 13% used SSRIs. Although BBs may shorten the QT interval and SSRIs may prolong it, our patients on both therapies were maintained on the same doses of their medications during all three EKG measurements and were exposed to the same ACT regimen as other patients throughout the study.

Prolongation of the QTc interval, which was the main endpoint of our study, was observed after the ACT chemotherapy regimen compared to baseline values. Chemotherapy agents act on the ion channels, particularly on sodium, potassium, and calcium channels, leading to the prolongation of cardiac cell action potentials (APs). AP prolongation, from the electrocardiographic viewpoint, translates into an increase in the QT interval. Individuals with this electrocardiographic alteration exhibit increased electrical heterogeneity of the myocardium, which results in the appearance of an early post-potential-triggered activity, which is an electrophysiological mechanism of electrical impulse generation that acts as a substrate for severe ventricular arrhythmias, such as *torsades de pointes* [8,17]. As demonstrated by Zamorano et al. [2], the patients most at risk of developing *torsades de pointes* are those with a QTc interval > 500 ms or a ΔQTc > 60 ms (i.e. change from baseline). In our series, nine patients (39, 14%) presented with a QTc interval > 500 ms, but none of them suffered life-threatening arrhythmias, which may be partially explained by the acute and subacute transient toxicity of chemotherapeutic agents.

Contrary to the findings of previous studies, our study did not observe any statistically significant differences in the secondary endpoints of QTdc or DTpTe [10,11,18]. Nousiainen et al. studied patients with non-Hodgkin lymphoma who underwent doxorubicin infusion and demonstrated an increased QTdc [10]. A similar QTdc result was also shown by Kuittinen et al. in patients with non-Hodgkin lymphoma who underwent chemotherapy with high doses of anthracycline and cyclophosphamide [18]. It is important to note that these two studies evaluated patients with non-Hodgkin lymphoma who received different chemotherapy regimens at higher doses than those used in breast cancer. Tanriverdi et al. showed an increase in QTdc in breast cancer patients with a chemotherapy regimen that was different than that used in our work and consisted of trastuzumab, epirubicin and cyclophosphamide [11]. QTdc and DTpTe more reliably reflect the heterogeneity of ventricular repolarization, but their electrocardiographic measurements can be technically difficult to perform when the QTc interval prolongation is mild due to lower doses of chemotherapy agents. The number of patients included in our study may have also contributed to our inability to demonstrate such differences. Another possible explanation is that changes in the QTc interval may occur earlier than changes in QTdc and DTpTe.

Troponin is a sensitive and specific biomarker of acute and subacute cardiotoxicity in patients undergoing chemotherapy with anthracyclines, as shown by Horacek et al [19]. Nevertheless, in patients with breast neoplasms undergoing the ACT regimen (doxorubicin, cyclophosphamide, and paclitaxel), this biomarker had not yet been studied. We demonstrated a significant increase in troponin, which is a marker of cell damage, at the end of the first (AC) phase and at the end of chemotherapy (after T) compared to baseline values. Cardinale et al. demonstrated that patients receiving high doses of doxorubicin had elevated serum troponin levels within the first 12–72 hours after starting treatment, with a decrease in the left ventricular ejection fraction after 7 months [20,21]. Markman et al. demonstrated that QTc prolongation was also associated with ventricular dysfunction in adulthood [22]; therefore, the acute changes in the ECG observed in our series as well as the elevation of troponin may reflect acute and subacute cardiotoxicity. We did not find a linear correlation between serum troponin dosage and QTc interval magnitude; however, changes in QTc interval magnitude can have other possible causes, such as a direct effect of chemotherapy on ventricular repolarization. The clinical significance of this finding requires further study.

Another important biomarker, BNP, shows conflicting data in the literature, and its elevation was demonstrated by Mavinkurve et al. to be a marker of cardiotoxicity [23], mainly in the context of hematological neoplasms. Among breast cancer patients treated with the doxorubicin, cyclophosphamide and paclitaxel chemotherapy regimen, levels of BNP have not yet been studied. In our study, we did not observe an elevation in BNP levels, likely because our patient population had structurally normal hearts and because the chemotherapy doses were lower than those used on patients with hematological neoplasms [10].

During the prospective follow-up period, we did not detect any of the secondary clinical endpoints, namely, CHF, cardiac arrhythmias, or death.

Several authors have demonstrated that the cardiotoxic effects of anthracyclines occur with cumulative doses of these chemotherapeutic agents [5,6]. Systolic dysfunction, the main complication associated with anthracyclines, can be observed at anthracycline doses of 400 mg/m^2^ or higher. In our study, we demonstrated that lower doses of doxorubicin (only 240 mg/m^2^) may cause acute and subacute cardiotoxicity, as shown by QTc prolongation and the rise in troponin levels. Early detection of QTc interval prolongation, which indicates the potential risk of malignant arrhythmias and sudden cardiac death, can provide an optimal monitoring of breast cancer patients undergoing an ACT regimen, both regarding the concentrations of electrolytes affecting the QT interval (potassium, calcium, and magnesium) and the introduction of medications that are known to prolong it. Further studies are needed to determine whether the administration of drugs such as BBs and angiotensin-converting enzyme (ACE) inhibitors can offer some protection during ACT treatment.

An open question in our study is whether these QT interval changes may disappear during long-term follow-up. Kuittinen et al. have demonstrated that the QTc interval prolongation and increase in QTdc observed in patients with non-Hodgkin lymphoma who received high doses of anthracycline and cyclophosphamide were reversible and recovered to normal values within 3 months after the end of chemotherapy [18].

Markman et al. showed that adult patients who survived pediatric neoplasms, were treated with anthracyclines and exhibited QTc interval prolongation in the acute phase also demonstrated ventricular dysfunction in adult life [22]. Given that this was a pediatric population, most of the tumors were probably of hematological origin, which are known to be treated with higher cumulative doses of anthracyclines. Whether our patients with breast neoplasms who displayed greater QT interval prolongation in the acute phase are at a higher risk of progressing to left ventricular dysfunction in the future merits further investigation.

Our study has some limitations. Although the number of patients is small, we also demonstrated that QT interval prolongation and elevated troponin levels occurred during the acute and subacute phases of ACT chemotherapy treatment. The criteria for arrhythmia documentation were based on clinical complaints and resting EKGs. A longer clinical follow-up duration and the use of Holter monitoring tools might increase detection of the clinical endpoints. The time for normalization of the QTc interval in the EKG of these patients with breast cancer is a limitation of our study and merits further investigation.

## Conclusions

In patients with breast neoplasms undergoing an ACT chemotherapy regimen, we observed prolongation of the QTc interval and elevated troponin levels during the treatment period; meanwhile, BNP serum levels were unchanged, and no alterations in QTdc or DTpTe were noted. There were no reports of clinical events, including arrhythmias, heart failure, angina, or death, during the study. The clinical significance of these findings requires additional observational and longitudinal studies with a longer follow-up period.

## Supporting information

S1 Table Values measured from the QTc interval. (XLSX)
